# Insulin stimulates SGLT2-mediated tubular glucose absorption via oxidative stress generation

**DOI:** 10.1186/s13098-015-0044-1

**Published:** 2015-05-24

**Authors:** Nobutaka Nakamura, Takanori Matsui, Yuji Ishibashi, Sho-ichi Yamagishi

**Affiliations:** Department of Pathophysiology and Therapeutics of Diabetic Vascular Complications, Kurume University School of Medicine, 67 Asahi-machi, Kurume, 830-0011 Japan

**Keywords:** SGLT2, Oxidative stress, Insulin, Diabetic nephropathy, AGEs

## Abstract

**Background:**

Ninety percent of glucose filtered by the glomerulus is reabsorbed by a sodium-glucose cotransporter 2 (SGLT2), which is expressed mainly on the apical membrane of renal proximal tubules. Since SGLT-2-mediated glucose reabsorption is enhanced under diabetic conditions, selective inhibition of SGLT2 has been proposed as a potential therapeutic target for the treatment of patients with diabetes. However, it remains unclear which diabetes-associated factors are involved in overexpression of SGLT2.

**Methods:**

Therefore, in this study, we examined whether insulin, high glucose, advanced glycation end products (AGEs), or H_2_O_2_ stimulated SGLT2 expression in human cultured proximal tubular cells, and then investigated the underlying molecular mechanisms.

**Results:**

High glucose or AGEs did not affect SGLT2 expression in tubular cells. Insulin significantly increased tubular SGLT2 level in a dose-dependent manner, whereas bell-shaped dose-response curves were observed for H_2_O_2_-treated cells. An anti-oxidant, *N*-acetylcysteine completely blocked insulin-induced up-regulation of SGLT2 as well as increase in glucose absorption by tubular cells. Furthermore, insulin dose-dependently increased reactive oxygen species generation in tubular cells.

**Conclusions:**

Our present study demonstrated that insulin could stimulate SGLT-2-mediated glucose entry into cultured proximal tubular cells via oxidative stress generation. Suppression of the insulin-induced overexpression of SGLT2 in tubular cells might be a novel therapeutic strategy for the treatment of diabetic nephropathy.

## Introduction

Diabetic nephropathy is a leading cause of end-stage renal disease, which could account for disabilities and high mortality rates in patients with diabetes [1, 2]. Diabetic nephropathy is characterized by functional and structural changes in the glomerulus such as glomerular hyperfiltration, thickening of glomerular basement membrane, and an expansion of extracellular matrix in the mesangial areas, which could ultimately progress glomerular sclerosis associated with an increased urinary excretion rate of albumin and renal dysfunction [2, 3]. Indeed, characteristic histological changes of diabetic nephropathy are diffuse and nodular glomerulosclerosis [2, 3]. However, it is supposed that changes within the tubulointerstitium are more important than glomerulopathy in terms of renal dysfunction in diabetic nephropathy [4, 5].

Ninety percent of glucose filtered by the glomerulus is reabsorbed by a low-affinity/high capacity sodium-glucose cotransporter 2 (SGLT2), which is expressed mainly on S1 and S2 segment of renal proximal tubules [6–8]. Since blockade of SGLT2 promotes urinary glucose excretion and resultantly improves hyperglycemia in an insulin-independent manner, SGLT2 inhibitors are now one of the widely used agents for the treatment of diabetes [9–11]. Furthermore, we have previously shown that increased glucose uptake into cultured renal proximal tubular cells via SGLT2 stimulates oxidative stress generation and resultantly potentiates the pro-apoptotic effects of advanced glycation end products (AGEs), senescent macroprotein derivatives formed acceleratedly under diabetes, on tubular cells [12, 13]. Therefore, blockade of SGLT2 could also be a therapeutic target for preventing tubular apoptosis and atrophy in diabetic nephropathy. SGLT2 levels in tubular cells harvested from the urine of diabetic subjects are increased compared with non-diabetic subjects [14]. However, which diabetes-associated factors are involved in SGLT2 overexpression in diabetic kidney remains unclear. Therefore, in this study, we examined whether insulin, high glucose, AGEs, or H_2_O_2_ stimulated SGLT2 expression in human cultured proximal tubular cells, and then investigated the underlying molecular mechanisms.

## Materials and methods

### Materials

Insulin, bovine serum albumin (BSA) (essentially fatty acid free and essentially globulin free, lyophilized powder), *N*-acetylcysteine (NAC), and poly-L-lysine were purchased from Sigma (St. Louis, MO, USA). D-Glucose and H_2_O_2_ from Wako Pure Chemical Industries Ltd. (Osaka, Japan). D-glyceraldehyde from Nakalai Tesque (Kyoto, Japan). Antibodies (Abs) directed against human SGLT2 and β-actin from Santa Cruz Biotechnology Inc. (Santa Cruz, CA, USA).

### Cells

Proximal tubular epithelial cells from human kidney were maintained in complete medium (basal medium supplemented with 5 % fetal bovine serum, 0.5 μg/ml hydrocortisone, 10 ng/ml human epidermal growth factor, 0.5 μg/ml epinephrine, 6.5 ng/ml triiodo-L-thyronine, 10 μg/ml transferrin, 5 μg/ml insulin, and GA-1000) according to the supplier’s instructions (Lonza Walkersville, Inc. Walkersville, MD, USA) [15]. Cells at 3-5 passages were used for the experiments. Insulin, H_2_O_2_, or other treatments were carried out in a serum-free basal medium containing 10 μg/ml transferrin and GA-1000.

### Preparation of AGEs-BSA

AGEs-BSA was prepared as described previously [16]. In brief, BSA (25 mg/ml) was incubated under sterile conditions with 0.1 M D-glyceraldehyde in 0.2 M NaPO_4_ buffer (pH 7.4) for 7 days. Then unincorporated sugars were removed by PD-10 column chromatography and dialysis against phosphate-buffered saline. Control non-glycated BSA was incubated in the same conditions except for the absence of reducing sugars.

### Western blot analysis

Tubular cells were treated with or without the indicated concentrations of insulin and H_2_O_2_, 11, 22 or 33 mM glucose, 100 μg/ml AGEs-BSA or non-glycated BSA in the presence or absence of 1 mM NAC. After 24 h, proteins were extracted from tubular cells with lysis buffer, and then separated by SDS-PAGE and transferred to nitrocellulose membranes as described previously [13]. Membranes were probed with Abs against SGLT2 or β-actin, and then immune complexes were visualized with an enhanced chemiluminescence detection system (Amersham Bioscience, Buckinghamshire, United Kingdom). Data were normalized by the intensity of β-actin-derived signals and related to the value of non-treated control cells.

### Assay for sodium-dependent glucose uptake

Tubular cells were treated with or without 50 ng/ml insulin in the presence or absence of 1 mM NAC for 24 h. Tubular cells were incubated with complete medium containing 100 μM 2-[N-(7-nitrobenz-2-oxa-1,3-diazol-4-yl)amino]-2-deoxy-D-glucose (2-NBDG, Peptide Institute Inc., Osaka, Japan), fluorescent derivative of glucose for 15 min. Then culture medium was removed and replaced with Hanks’ balanced salt solution, and fluorescence intensity in the cells was analyzed in an ARVO fluorescent plate reader (PerkinElmer, Inc., Winter Street Waltham, MA, USA) as described previously [13].

### Measurement of reactive oxygen species (ROS) generation

Intracellular formation of ROS was detected using a fluorescent probe carboxy-H_2_DFFDA (Life Technologies Japan, Tokyo, Japan) as described previously [17]. In brief, 96-well plates (FALCON, New York, NY, USA) were coated with 0.01 % poly-L-lysin for 30 min at room temperature. Then tubular cells were seeded into the well, and incubated with 0.1 % dimethyl sulfoxide (DMSO) in the presence or absence of 10 μM carboxy-H_2_DFFDA for 1 h. Then the cells were washed with phosphate-buffered saline, and treated with or without the indicated concentrations of insulin. After 24 min, intracellular ROS generation was measured with an ARVO X3 fluorescent plate reader (PerkinElmer Japan, Yokohama, Japan). ROS production was calculated by subtracting the fluorescence for cells pre-incubated with DMSO only from that with carboxy-H_2_DFFDA. Under cell-free conditions, 10 μM carboxy-H_2_DFFDA was also incubated with the indicated concentrations of H_2_O_2_ for 24 min, and then the fluorescence was measured.

### Statistical analysis

All values are presented as mean ± standard deviation. One-way analysis of variance followed Tukey’s test or student’s *t*-test was performed for statistical comparisons; p-values of less than 0.05 were considered significant.

## Results

### Effects of insulin, high glucose, AGEs, or H_2_O_2_ on SGLT2 expression

**Fig. 1:**
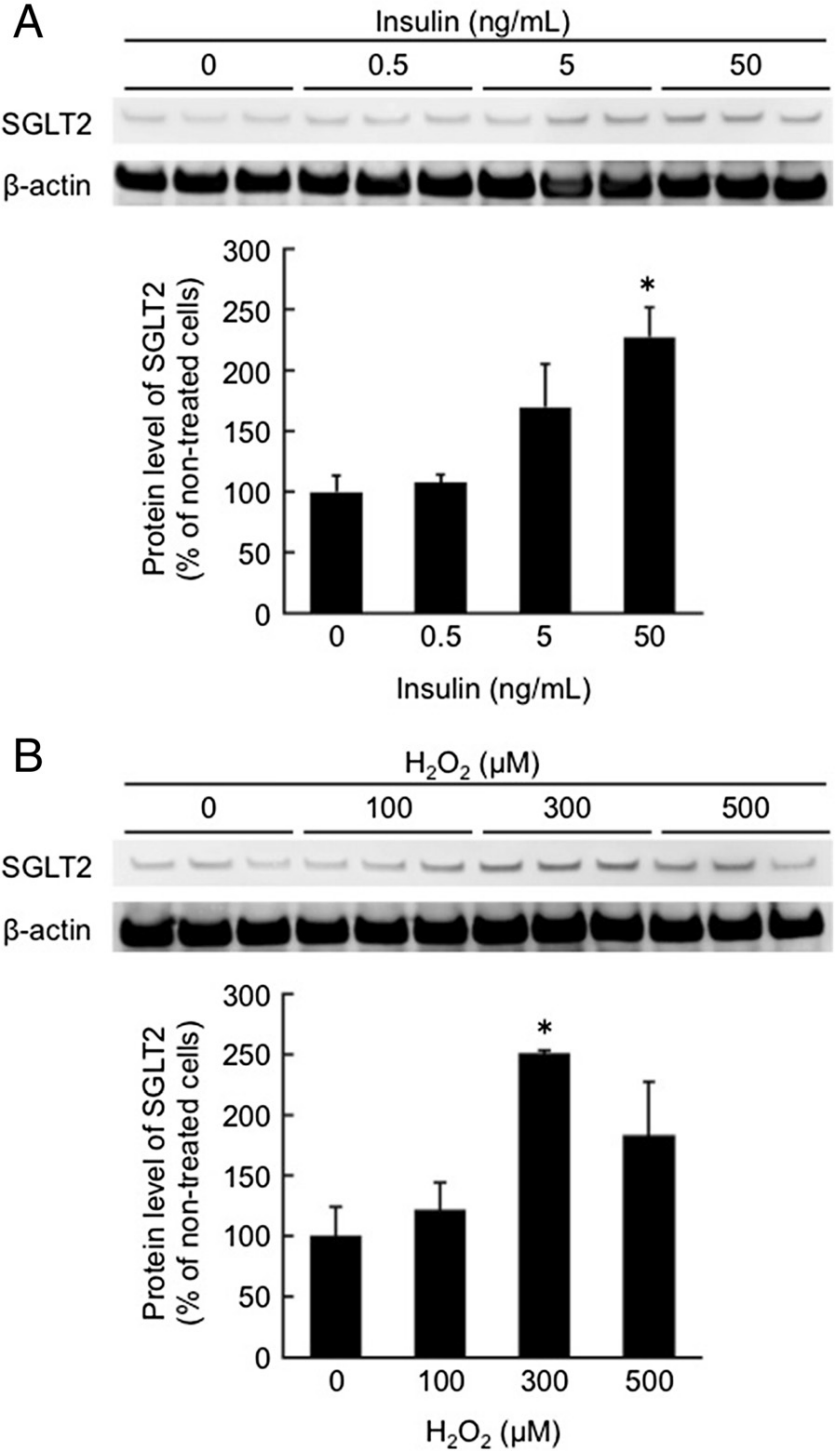
Effects of insulin (**a**) and H_2_O_2_ (**b**) on SGLT2 expression in tubular cells. Tubular cells were treated with or without the indicated concentrations of insulin and H_2_O_2_. After 24 h, proteins were extracted from tubular cells with lysis buffer, and then separated by SDS-PAGE and transferred to nitrocellulose membranes. Membranes were probed with Abs against SGLT2 or β-actin, and then immune complexes were visualized with an enhanced chemiluminescence detection system. Each upper panel shows the representative bands of western blots. Each lower panel shows the quantitative data. Data were normalized by the intensity of β-actin-derived signals and related to the value of non-treated control cells. N = 3. *, p < 0.05 compared with the values of non-treated cells

We first examined whether insulin, high glucose, AGEs, or H_2_O_2_ stimulated SGLT2 expression in cultured proximal tubular cells. High glucose up to 33 mM or 100 μg/ml AGEs, comparable levels with plasma concentrations under diabetic situations [18], did not affect SGLT2 expression in tubular cells (data not shown). However, as shown in Figs. 1a and b, insulin significantly increased tubular SGLT2 expression level in a dose-dependent manner, whereas bell-shaped dose-response curves were observed for H_2_O_2_-treated cells. Since maximum response was obtained in 50 ng/ml insulin-treated cells (Fig. 1a), we chose the condition of 50 ng/ml insulin for the following experiments.

### Effects of NAC on insulin-induced SGLT2 expression and glucose uptake by tubular cells

**Fig. 2:**
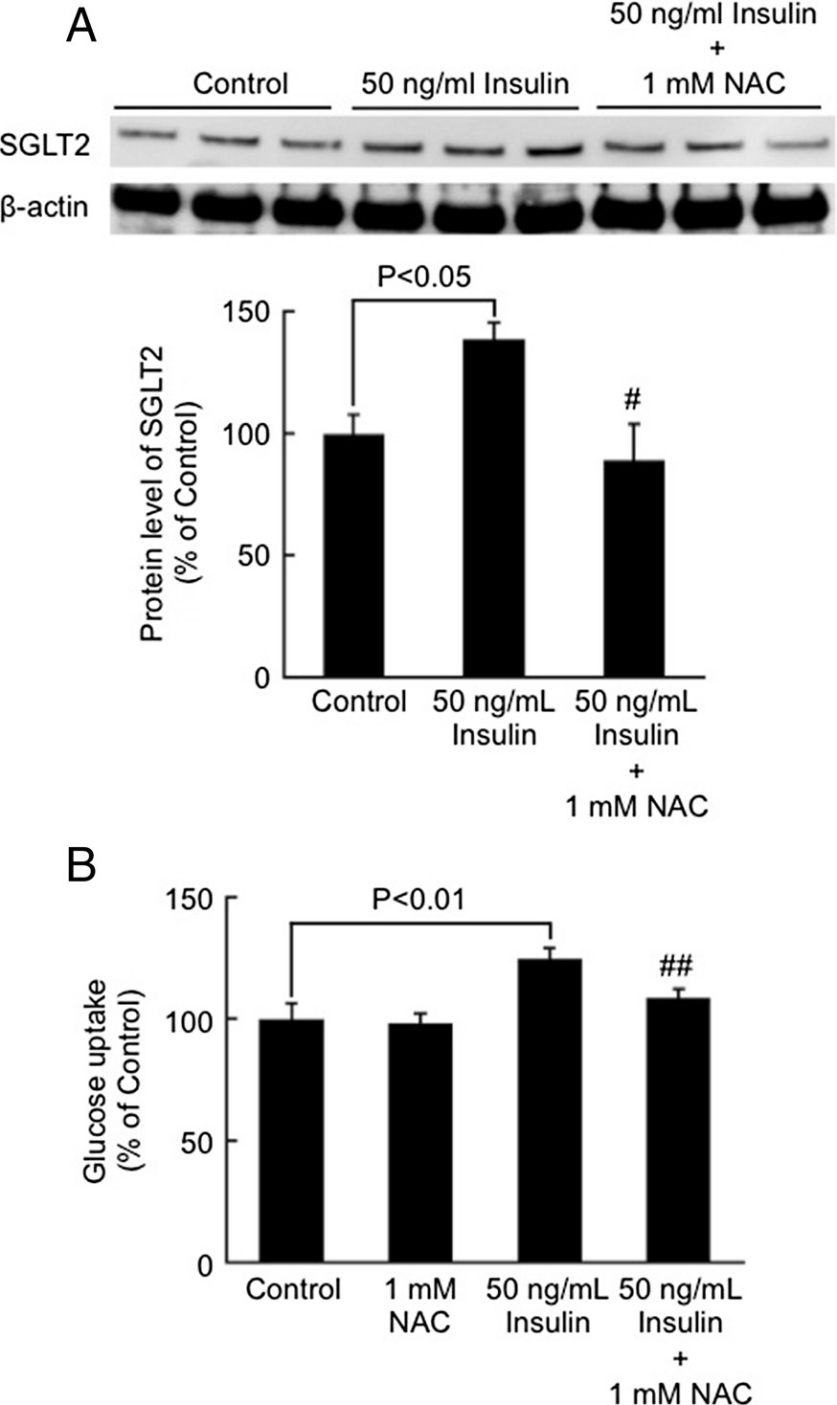
Effects of NAC on insulin-induced SGLT2 expression (**a**) and glucose uptake (**b**) by tubular cells. Tubular cells were treated with or without 50 ng/ml insulin in the presence or absence of 1 mM NAC for 24 h. **a** SGLT2 protein level was analyzed with western blot analysis. Upper panel shows the representative bands. Lower panel shows the quantitative data. N = 3. **b** Tubular cells were incubated with complete medium containing 100 μM 2-NBDG for 15 min. Then fluorescence intensity in the cells was analyzed. N = 6. Data of two independent experiments were combined. # and ##, p < 0.05 and p < 0.01, respectively compared with the values of 50 ng/ml insulin-treated cells

We next studied the effects of an anti-oxidant NAC on insulin-induced SGLT2 expression and glucose uptake by tubular cells. As shown in Fig. 2a, 1 mM NAC completely blocked the insulin-induced up-regulation of SGLT2 level in tubular cells. Furthermore, 50 ng/ml insulin significantly increased glucose entry into tubular cells, which was also completely prevented by the treatment with 1 mM NAC (Fig. 2b).

### Effects of insulin on ROS generation in tubular cells

**Fig. 3:**
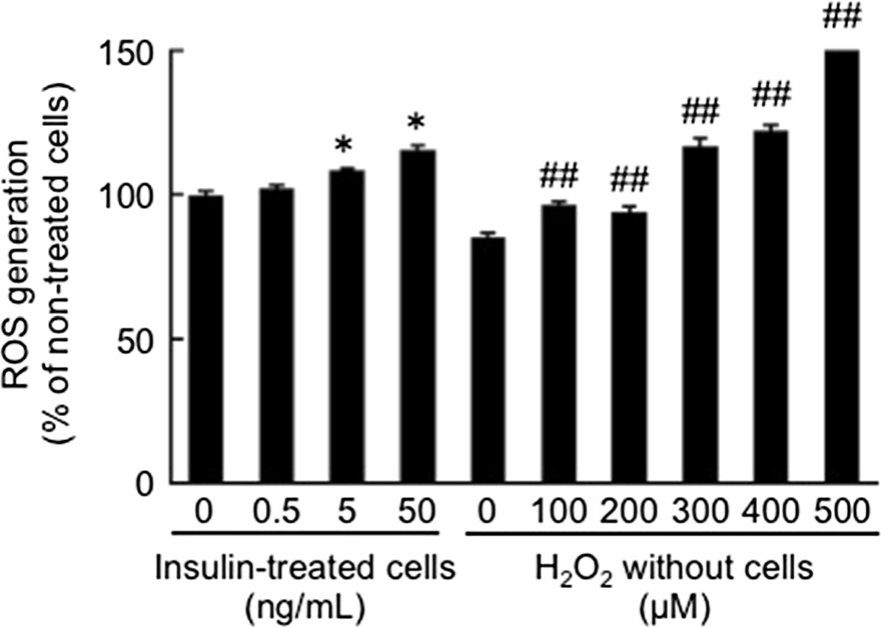
Effects of insulin on ROS generation in tubular cells. Tubular cells were incubated with 0.1 % DMSO in the presence or absence of 10 μM carboxy-H_2_DFFDA for 1 h. Then the cells were treated with or without the indicated concentrations of insulin. After 24 min, ROS generation was measured. Under cell-free conditions, 10 μM carboxy-H_2_DFFDA was incubated with the indicated concentrations of H_2_O_2_ for 24 min, and then the fluorescence was also measured. N = 6. *, p < 0.05 compared with the values of non-treated cells. ##, p < 0.01 compared with the values under cell-free conditions without H_2_O_2_

We investigated the effects of insulin on ROS generation in tubular cells. As shown in Fig. 3, insulin dose-dependently increased ROS generation in tubular cells. ROS production in tubular cells elicited by 50 ng/ml insulin was comparable with that of 300 μM H_2_O_2_.

## Discussion

In this study, we found that (1) 50 ng/ml insulin or 300 μM H_2_O_2_ significantly increased SGLT2 expression level in cultured proximal tubular cells, (2) an anti-oxidant NAC completely inhibited up-regulation of SGLT2 level in, and subsequently blocked the increase of glucose uptake by, 50 ng/ml insulin or exposed tubular cells, (3) 50 ng/ml insulin-evoked ROS generation in tubular cells was comparable with that of 300 μM H_2_O_2_, and (4) magnitude of increase in SGLT2 protein induced by 50 ng/ml insulin was similar to that by 300 μM H_2_O_2_. These observations suggest that insulin might stimulate SGLT-2-mediated glucose entry into cultured proximal tubular cells via oxidative stress generation.

There is some controversy about the expression level of SGLT2 in the diabetic kidney of type 1 diabetic animals; SGLT2 were decreased, unchanged or increased in streptozotocin-induced diabetic rats [19–22]. However, in contrast to type 1 diabetic animals, increased SGLT2 expression was consistently observed in the kidney of type 2 diabetic animals [23–25] and in tubular cells harvested from the urine of type 2 diabetic subjects [17], the latter of which was correlated with glucose reabsorption capacity in these patients. Therefore, insulin resistance and resultant hyperinsulinemia may contribute to SGLT2 overexpression in the kidney of type 2 diabetes. The ability of insulin to stimulate sodium reabsorption in proximal tubules is preserved in insulin-resistant subjects despite resistance to insulin metabolic effects [26]. So, the selective insulin resistance might be involved in SGLT2 induction under hyperinsulinemic conditions.

In the present study, although high glucose or AGEs have been reported to stimulate ROS generation in tubular cells [12, 13, 17], neither of them increased tubular SGLT2 expression (data not shown). High glucose or AGEs has been reported to induce apoptotic cell death of cultured proximal tubular cells [12, 13, 17], whereas insulin has anti-apoptotic properties in tubular cells and stimulates proliferation of this cell type [27, 28]. Furthermore, while high level of ROS is toxic to various types of cells, including proximal tubular cells [29–31], relatively low level of intracellular ROS could function as a second messenger in signaling cascades involved in gene expression [30–32]. Indeed, insulin-induced ROS generation has been coupled with its action in insulin-sensitive cells [30]. Therefore, the action of ROS on SGLT2 expression in tubular cells might also depend on its concentration. ROS generation evoked by high glucose or AGEs may be higher than 300 μM H_2_O_2_ and toxic to cells, which might partly explain the reason why these two agents cannot induce tubular SGLT2 expression. In this study, (1) SGLT2 level induced by 500 μM H_2_O_2_ was less than that by 300 μM H_2_O_2_ (Fig. 1b), and (2) total protein amounts obtained from 500 μM H_2_O_2_-exposed tubular cells were decreased to about 70 % of those of non-treated controls or 300 μM H_2_O_2_-exposed cells, thus supporting the concept that toxic level of ROS could affect SGLT2 expression in tubular cells.

We have previously shown that SGLT2-mediated glucose overload in tubular cells could augment the cells’ susceptibility toward pro-apoptotic effects of AGEs via overexpression of receptor for AGEs (RAGE) [13]. Furthermore, we, along with others, have recently found that empagliflozin, an inhibitor of SGLT2, suppresses oxidative, inflammatory and fibrotic reactions in the kidney and aorta of diabetic rats partly via suppression of the AGEs-RAGE axis [21, 33]. Apoptosis of proximal tubular cells plays a central role in tubular atrophy and atubular glomeruli of diabetic nephropathy [34, 35], which are most closely correlated with declining creatinine clearance in patients with diabetes [4, 5]. Given the pathological role of the AGEs-RAGE axis in tubular cell apoptosis [12, 13, 17], blockade of insulin-induced SGLT2 overexpression may not just improve hyperglycemia by promoting urinary glucose excretion, but could also directly inhibit glucotoxicity to proximal tubular cells, thus protecting against tubulointerstitial damage in diabetic nephropathy.

### Limitations

We did experiments of Figs. 1a, b and 2a separately. So the 50 ng/ml column in Figs. 1a and 2a was not the same data. Since exposure time of an enhanced chemiluminescence detection system in each experiment differed, we were not able to show the data with the same units. This was a reason why we showed the data of non-treated cells in each experiment as a control. The physiological concentration of insulin in humans is 0.5-5 ng/ml. Oral administration of NAC for the treatment of acetaminophen poisoning obtained a plasma level of NAC at 10 μM [36]. Therefore, the concentration of insulin (50 ng/ml) and NAC (1 mM) having biological effects on tubular cells used in the present experiments may be in the superphysiologic range. This study was only analyzed in cell culture, not investigated about animal models and human. Therefore, further study is needed to clarify whether hyperinsulinemia may contribute to SGLT2 overexpression in animal model or human diabetic kidneys.

## Conclusions

Our present study demonstrated that insulin could stimulate SGLT-2-mediated glucose entry into cultured proximal tubular cells via oxidative stress generation. Suppression of the insulin-induced overexpression of SGLT2 in tubular cells might be a novel therapeutic strategy for the treatment of diabetic nephropathy.

## Abbreviations

Abs: Antibodies
AGEs: Advanced glycation end products
BSA: Bovine serum albumin
DMSO: Dimethyl sulfoxide
NAC: *N*-acetylcysteine
2-NBDG: 2-[N-(7-nitrobenz-2-oxa-1,3-diazol-4-yl)amino]-2-deoxy-D-glucose
ROS: Reactive oxygen species
SGLT2: Sodium-glucose cotransporter 2
RAGE: Receptor for AGEs
